# Acute Psychosis and COVID-19 Infection: Psychiatric Symptoms in Hospitalized Patients

**DOI:** 10.7759/cureus.18121

**Published:** 2021-09-20

**Authors:** Sana Elham Kazi, Selina Akhter, Divya Periasamy, Farzana Faruki, Rana Tahir

**Affiliations:** 1 Psychiatry, Brookdale University Hospital Medical Center, New York City, USA; 2 Psychiatry and Behavioral Sciences, Brookdale University Hospital Medical Center, New York City, USA

**Keywords:** pneumonia, schizophrenia, diabetes, acute respiratory failure, psychiatric symptoms, neurocognitive symptoms, psychosis, psychiatric illnesses, covid-19, sars-cov-2

## Abstract

The severe acute respiratory syndrome coronavirus 2 (SARS-CoV-2) infection has resulted in various medical and psychosocial consequences globally. Respiratory infections are common among patients infected with the SARS-CoV-2 virus, the causative virus of coronavirus disease 2019 (COVID-19). However, various psychiatric and neurocognitive symptoms and sequelae of COVID-19 have been reported as well.

This study aimed to describe two clinical case reports of patients with no prior history of psychiatric illnesses admitted to the psychiatric inpatient unit with acute onset of psychosis. A 49-year-old woman with no past medical history and no past psychiatric history was admitted to the inpatient psychiatric unit with suicidal ideation and was noted to have acute psychosis. A 56-year-old woman with a history of hypertension with no past psychiatric history was admitted to the hospital with acute hypoxic respiratory failure secondary to COVID-19 pneumonia and was noted to have acute psychosis.

Various psychiatric and neurocognitive symptoms and sequelae of COVID-19 have been reported. However, the pathophysiology, direct biological effects of the disease, treatment modalities, worsening of symptoms due to various medications, and other long-term sequelae are not fully understood. Therefore, clinicians should be mindful of neuropsychiatric symptoms and conduct a detailed history and physical examination on all patients presenting with psychiatric symptoms in the context of COVID-19. It is also essential to assess for signs and symptoms of delirium in patients presenting with neuropsychiatric symptoms. Further research is needed to identify the etiology, predisposing factors, exacerbating or precipitating factors contributing to neuropsychiatric symptoms associated with infection with the SARS-CoV-2 virus. In addition, the pathophysiology contributing to these symptoms and pharmacological interventions for managing these sequelae need to be evaluated.

## Introduction

The global coronavirus disease 2019 (COVID-19) pandemic has resulted in various medical and psychosocial consequences. The severe acute respiratory syndrome coronavirus 2 (SARS-CoV-2) that causes COVID-19 impacts multiple organ systems. Extensive population-based cross-sectional and longitudinal data suggest that at least one-third of SARS-CoV-2 infections are asymptomatic [1]. 

Among symptomatic patients with COVID-19, various symptoms, including systemic symptoms such as fever, fatigue, myalgia, rigors, arthralgia, and rash and respiratory symptoms such as cough (dry or productive), dyspnea, chest pain, hemoptysis, and wheeze have been reported [2,3]. 

In addition to constitutional and respiratory symptoms, there is growing evidence of increased incidence of various psychiatric and neurocognitive symptoms of COVID-19. Neurological symptoms include loss of smell or taste, visual disturbances in mild disease, myalgias, ataxia, and encephalopathy in moderate disease. In severe disease, meningoencephalitis, seizures, stroke, or coma can be noted. Psychiatric symptoms include depression, anxiety, post-traumatic stress disorder, and exacerbation of co-existing psychiatric illnesses. 

In this study of two cases, we describe two clinical cases of patients with no prior history of psychiatric illnesses admitted to the psychiatric inpatient unit with acute onset of psychosis. 

## Case presentation

Case 1

A 49-year-old woman with no past medical or psychiatric history was admitted to the inpatient psychiatric unit with suicidal ideation. For the past two weeks, the patient's family members reported that the patient expressed thoughts that the world was ending. She was not sleeping, was increasingly agitated and not eating, and had an 8-pound weight loss. She had expressed thoughts of wanting to jump in front of the train, feeling morbidly guilty and hopeless. She also reported feeling helpless with multiple deaths in the city due to COVID-19, and the situation reminded her of her grandmother's death due to cancer. She also had paranoid delusions with nightmares and felt responsible for her grandmother's death due to cancer about 40 years ago. 

She was a high school graduate and worked as a hairdresser and cleaner in a hair salon for 15 years. She stopped working a month ago due to COVID-19 and reported being stressed about this. She lived in an apartment with her three children, a 25-year-old son, a 22-year-old son, and a 12-year-old daughter. She had periodic contact with the father of her children, who lives in a different state. She also broke off a one-year romantic relationship with her boyfriend about two months ago. She denied a history of sexual or physical abuse as a child. She had no past psychiatric history or suicide attempts or substance use or criminal history and no family history of depression. 

She also had a three-week history of on and off cough with a reduced appetite and anosmia but denied fever, malaise, and body aches. On examination, she was guarded, withdrawn, disheveled, and depressed with constricted affect. She was alert and oriented to time, place, and person with no focal neurological deficits. Besides, her memory and concentration were intact with a goal-directed thought process, however, with limited insight and judgment. Bloodwork showed normal white cell and platelet count, hemoglobin and hematocrit, and normal glucose and electrolytes, kidney, liver, and thyroid function tests. Urinalysis showed moderate leucocytes, trace blood, trace protein, positive for ketones and negative for nitrite and glucose. Urine microscopy showed few bacteria, 25-50 white cells, 5-10 red cells, moderate epithelial cells, and moderate mucus threads. Drug toxicology screen and lithium, carbamazepine, and valproic acid levels were negative. She tested positive for SARS-CoV-2 on admission. Oral aripiprazole 5 mg was started for psychotic symptoms, and the dose was titrated up to 7 mg. She was also started on oral escitalopram 10 mg for depressed mood, lorazepam 2 mg for anxiety, and oral mirtazapine 15 mg for poor sleep and poor appetite. Oral azithromycin and oral Hydroxychloroquine were started for SARS-CoV-2 infection for five days. Chest x-ray, EKG, and CT head were unremarkable.

On day four of hospital admission, she continued to express suicidal ideation and wanted to starve herself. She remained withdrawn, guarded, with poor appetite and sleep. She also had a brief catatonic episode of muteness for less than a minute. Subsequently, she got up from her bed, was agitated, and tried to run out of her isolation room. Repeat blood work showed normal white count, normal hemoglobin, platelets, electrolytes, and renal and liver function tests. She was given haloperidol, diphenhydramine, and lorazepam. Aripiprazole was discontinued and she was started on oral metoprolol for hypertension and oral olanzapine 7.5 mg for psychosis, poor sleep, and poor appetite, and the dose was slowly titrated up to 20 mg. With this regimen, clinical improvement was noted from admission days four to 15, and the patient’s depression, appetite, and behavior improved, with no suicidal ideation. There was a plan to send the patient home on day 16. However, on the scheduled discharge day, she reported a loss of appetite with paranoid behavior and she complained that she would be investigated and arrested if she left the hospital because she had COVID-19, so she did not want to leave the hospital. On examination, the patient was alert and oriented but showed guarded behavior and limited judgment. Subsequently, the olanzapine dose was increased to 25 mg, and oral aripiprazole 2 mg was re-started. From days 17 to 21, she showed clinical improvement, mood improvement, and improvement in her thoughts with no more paranoid behavior and no suicidal or homicidal ideation. She was subsequently discharged home on day 21 on olanzapine 25 mg, escitalopram 20 mg, and mirtazapine 15 mg, and metoprolol 25 mg. She had outpatient follow-ups with a psychiatrist after discharge and did not exhibit paranoid behavior or manic episodes. Olanzapine was stopped two months later due to akathisia as an adverse effect. Eight months after initial hospitalization, she was re-admitted to the inpatient psychiatric unit for grandiose, erratic, disorganized behavior at home, verbal aggression at work, and violently confronting strangers on the street. She was treated for 20 days in the inpatient psychiatric unit and diagnosed with bipolar disorder (current episode manic) with psychosis. She was then discharged on oral risperidone 3 mg twice daily, valproate 500 mg twice daily for mood, and metoprolol 25 mg once daily for hypertension.

Case 2

A 56-year-old African American woman with a history of hypertension and not on any medications at home with no past psychiatric history was admitted to the hospital with acute hypoxic respiratory failure secondary to COVID-19 pneumonia. 

On arrival to the emergency room, she was noted to be hypoxic on room air and required high flow oxygen therapy, and tested positive for SARS-CoV-2 infection. She reported symptoms of shortness of breath, chills, cough for two weeks. Her husband was sick at home for two weeks, and her boss was also ill at work. Physical examination revealed the patient was in acute distress with labored breathing and was speaking in short sentences. She was alert and oriented with a normal neurological exam with no focal neurological deficits. Urinalysis was not performed. Inflammatory markers including ferritin, C-reactive protein, sedimentation rate, and D-dimer were elevated. Chest x-ray showed extensive airspace consolidation throughout both lungs, consistent with extensive bilateral pneumonia. Chest CT angiogram revealed bilateral diffuse pulmonary infiltrate with mild cardiomegaly, no pulmonary embolus, and no pleural effusion.

She was then admitted to the inpatient unit for acute hypoxic respiratory failure and treated with vitamin C, vitamin D, zinc, one dose of monoclonal antibody, tocilizumab, dexamethasone for 10 days, and remdesivir for five days. In addition, she was treated with full-dose of enoxaparin for elevated D-dimer levels. She was also diagnosed with new-onset type 2 diabetes mellitus with a hemoglobin A1C of 14.4 and was started on insulin. Throughout this hospital admission, her oxygen requirement progressively decreased as she was weaned off from high flow nasal cannula to nasal cannula and then room air. 

She exhibited altered mental status with bizarre delusions and agitated behavior during this hospital admission. She claimed that her boss had poisoned her and that the blood work would prove it. Her son provided collateral history and mentioned that her boss gave her antibiotics, possibly azithromycin and ivermectin, supposedly obtained from Tanzania. She took the antibiotics for a week and stopped a few days ago. Few days before the hospital admission, she experienced hallucinations and bizarre delusions and talked about "numbers on the wall." There was no prior history of psychiatric illness, no history of similar complaints in the past, no history of suicidal/homicidal ideations, no family history of mental illness, no family history of suicide, no drug use, and no other instances of paranoid behavior. She is a teacher at a daycare and the primary caregiver for her boyfriend and lives with her boyfriend and has three children, two sons and one daughter. 

She mentioned that the board of education sent her as an undercover to observe and investigate and shut down this hospital. She also reported visual and auditory hallucinations, seeing numbers all over the walls, and the spirit of God was talking to her. She also reported mania-like symptoms with inflated self-esteem, easy distractibility, circumstantial thought process, rapid speech, racing thoughts, poor sleep at night, and despite minimally sleeping, she felt energetic before coming to the hospital. The plan was to start the patient on antipsychotics for her acute psychosis; however, these were initially placed on hold due to a prolonged QT interval of 550 milliseconds on EKG on admission. Urine drug screen, rapid plasma reagin (RPR) test for syphilis, and HIV testing were negative. Two samples of blood culture showed no growth after five days of incubation. CT head was normal. She exhibited paranoid behavior, believing that she was raped during this hospital stay. She thought the hospital had killed the patient next to her bed, and the staff was laughing at her. She was given oral aripiprazole 5 mg and intramuscular olanzapine 5 mg and was then discharged to an outside facility for further psychiatric management due to COVID-19. There was no follow-up after discharge. 

## Discussion

COVID-19 affects various organ systems with variable symptoms based on disease severity (Table 1) [4]. Various psychiatric and neurocognitive symptoms and sequelae of COVID-19 infection have been reported. Symptoms include fatigue, deconditioning, memory, cognitive, emotional, and behavioral problems [5]. 

**Table 1:** Symptoms of COVID-19 Infections Based on Disease Severity

Organ Systems	Mild COVID-19 Infection	Moderate COVID-19 Infection	Severe COVID-19 Infection
Upper and lower respiratory	Cough (dry and productive), sore throat, rhinorrhea, sneezing	Dyspnea, pneumonia moderate hypoxemia	Severe hypoxemia, acute respiratory distress syndrome (ARDS), respiratory failure
Neurological	Hyposmia-anosmia, hypogeusia-ageusia, visual disturbance, fatigue, daytime sleepiness	Headaches, nausea and vomiting, dizziness, myalgia, ataxia, encephalopathy	Cerebrovascular disease, seizures, meningoencephalitis, neuropathy, Guillain Barre syndrome, neurogenic, ARDS, coma
Gastrointestinal	Nausea, vomiting, diarrhea, heartburn	Loss of appetite, abdominal pain, bloating	Gastrointestinal bleeding
Cardiac	Chest pain, arrhythmia, sinus tachycardia	Cardiac inflammation	Cardiomyopathy, acute heart failure
Renal	Proteinuria, hematuria	Acute renal injury	Renal failure
Vascular	Blood coagulation	Arterial or venous thromboembolism	Pulmonary embolism, large vessel occlusions, disseminated intravascular coagulation
Psychiatric	Depressed mood, anxiety, insomnia, anger, fear	Depression, post-traumatic stress disorder	Exacerbation of neurological or psychiatric disorders

Psychosis related to COVID-19 is the most probable cause for both these patients described above with the possibility of a manic episode with psychosis in case 2. However, there was no follow-up, and hence a conclusive diagnosis cannot be reached. A systematic review of 33 studies evaluated the neuropsychological and psychiatric sequelae of community-dwelling patients recovering or recovering from COVID-19. These patients had high rates of depression, anxiety, fatigue, sleep disruption, and post-traumatic stress. In addition, a significant portion of these patients who survived COVID-19 showed poor cognitive performance in attention, executive function, and memory [6].

Case 1 also had identifiable stressors, including unemployment due to the COVID-19 pandemic and a recent breakup with her boyfriend. Psychiatric illness in COVID may occur from various factors, including medical condition, functional disability, stressors including anxiety and fear associated with the disease, and psychosocial and environmental factors including social distancing and restrictions. 

As part of the Coroner National Registry Study, data from 153 patients admitted to the hospitals presenting with neurological and neuropsychiatric features within the United Kingdom was outlined [7]. Out of these 153 patients, 125 patients had complete data and 62% of these patients presented with a cerebrovascular event, and 31% exhibited altered mental status. Among those with altered mental status, 18% had encephalitis, 23% had encephalopathy, and 59% had a neuropsychiatric disorder. Of the 23 neuropsychiatric cases, 43% had new-onset psychosis, 26% had a neurocognitive (dementia-like) syndrome, and 17% had an affective disorder. 

Survivors of COVID-19 appear to be at increased risk of psychiatric sequelae, and a psychiatric diagnosis might be an independent risk factor for COVID-19 based on a cohort study using data from 62,354 COVID-19 cases in the United States [8]. Patients with schizophrenia spectrum disorder have several risk factors, including health-related and socioeconomic risks, potentially putting them at a higher likelihood of negative consequences [9].

Several case reports have been published on the new onset of psychotic symptoms in the context of COVID-19 infection. However, the pathophysiology, direct biological effects of the disease, treatment modalities, worsening of symptoms due to various medications, long term sequelae are not fully understood. Structured delusions with confusion were the most frequent psychiatric manifestations observed in the COVID-19 patients based on a retrospective descriptive study [10].

In addition to pandemic-related psychological distress, various mechanisms contributing to neuropsychiatric sequelae due to COVID-19 infection have been described. These include viral infiltration into the central nervous system, cytokine network dysregulation, peripheral immune cell transmigration, and post-infectious autoimmunity [11].

There is an increase in inflammatory markers in SARS-CoV-2 infection, indicating that neuroimmune mechanisms may be involved. Cytokine storm and increased immune responses are noted in COVID-19. There is an increase in proinflammatory cytokines, including interleukin (IL) 1, IL-6, IL-10, and tumor necrosis factor (TNF)-α. Cytokines penetrate the blood-brain barrier resulting in widespread neuronal damage, and this is associated with poor prognosis. Widespread inflammation has been noted in the olfactory bulbs and medulla oblongata, reflecting the loss of smell and many of the neurological symptoms of COVID-19. SARS-CoV-2 has also been detected in the brain with microglial activation and cytotoxic T lymphocyte infiltration. The SARS-CoV-2 virus enters through the angiotensin-converting enzyme-2 receptors and damages endothelial cells. Activated microglia secrete inflammatory mediators results in increased glutamate and N-methyl-d-aspartate (NMDA), resulting in difficulty learning, memory, hallucinations, and nightmares [12-15]. Of note, case 2 was noted to have elevated inflammatory markers. 

In the differential diagnosis, the other stronger consideration is delirium, secondary to urinary tract infection (UTI) or hypoxemia associated with acute respiratory failure. Both patients did not report any urinary symptoms. Urinalysis and urine microscopy in case 1 showed few bacteria with moderate leukocytes and no nitrites, possibly a first void urine sample rather than clean catch urine. However, no urine culture was performed. UTIs are common in patients with diabetes, and case 2 has new-onset diabetes. Urinalysis or culture was not performed in case 2. There is a 19.4% prevalence of UTIs in delirium, 11.2% in dementia, 21.7% in nonaffective psychotic disorders, and 17.8% in mood disorders [16]. Delirium is usually diagnosed with the help of instrumental tools, including the confusion assessment method (CAM) or CAM-intensive care unit (ICU). Based on the patients' history and physical exam, delirium does not appear to be a likely cause. 

The third possibility for acute psychosis is the probable intake of unknown medications, possibly ivermectin and azithromycin, by case 2. It is not known if case 2 took ivermectin before the hospital admission. Neurologically severe adverse events have been reported using Ivermectin, including encephalopathy, confusion, stupor, or coma and symptoms of neurotoxicity including lethargy, drooling, tremors/seizures, inability to stand, disorientation, and coma [17]. In addition, delirium associated with Ivermectin toxicity has been reported; however, hardly any other reports of acute psychosis associated with the use of ivermectin have been reported [18]. 

Regarding azithromycin use, case 2 reported possibly taking it before hospital admission, and case 1 was given azithromycin during the hospital admission. Azithromycin acts against Zika and Ebola viruses by interfering with their protein synthesis and attains high and sustained concentration in brain tissue [19]. Few neurological adverse events have been reported using azithromycin, and severe neuropsychiatric adverse effects such as delirium have rarely been reported [20]. Visual and auditory hallucinations, multiple partial complex seizures, severe headaches, and recurrent cortical blindness have been documented using azithromycin [21]. 

The other factor to consider is steroid-induced psychosis. Case 2 was started on dexamethasone for COVID-19-associated pneumonia. Neuropsychiatric side effects of glucocorticoids have been reported ranging from mild irritability or euphoria to acute psychosis with suicidal ideation [22]. However, she reported psychiatric symptoms before starting the glucocorticoid, dexamethasone, and hence steroid-induced psychosis can be ruled out. 

The chance of psychosis related to hydroxychloroquine is also unlikely in the patient, case 1. Psychosis induced by hydroxychloroquine has been described only in a few case reports and is expected to be quite rare [23]. Psychosis after chloroquine use has been reported more frequently than hydroxychloroquine [24]. However, case 1 had symptoms of paranoid behavior before starting hydroxychloroquine. 

## Conclusions

In conclusion, this study presents the importance of paying attention to neuropsychiatric complaints in patients with COVID-19. In cases 1 and 2, the previously healthy patients with no prior psychiatric history became acutely psychotic in the context of COVID-19. Therefore, we recommend that clinicians should be mindful of neuropsychiatric symptoms and conduct a detailed history and physical examination on all patients presenting with psychiatric symptoms in the context of COVID-19. It is also essential to assess for signs and symptoms of delirium in patients presenting with neuropsychiatric symptoms. Further research is needed to identify the etiology, predisposing factors, exacerbating or precipitating factors, mode, and mechanisms contributing to neuropsychiatric sequelae associated with infection with SARS-CoV-2 virus and pharmacological interventions to manage these sequelae. The limitation of the current case series is the unknown contribution of psychosocial factors, other co-morbidities, including diabetes, and other prescription and non-prescription medications in the development of neuropsychiatric complaints. 
